# Genetic Markers of Cardiovascular Disease in Rheumatoid Arthritis

**DOI:** 10.1155/2012/574817

**Published:** 2012-08-02

**Authors:** Luis Rodríguez-Rodríguez, Raquel López-Mejías, Mercedes García-Bermúdez, Carlos González-Juanatey, Miguel A. González-Gay, Javier Martín

**Affiliations:** ^1^Department of Rheumatology, Hospital Clínico San Carlos, c/Profesor Martín Lagos s/n, 28040 Madrid, Spain; ^2^Instituto de Parasitología y Biomedicina López-Neyra, CSIC, Parque Tecnológico de Ciencias de la Salud, Avenida del Conocimiento s/n, Armilla, 18100 Granada, Spain; ^3^Department of Rheumatology, Hospital Universitario Marqués de Valdecilla, IFIMAV, Avenida Herrera Oria s/n, 39011 Santander, Spain; ^4^Cardiology Division, Hospital Xeral-Calde, c/Dr. Ochoa s/n, 27004 Lugo, Spain

## Abstract

Cardiovascular (CV) disease is the most common cause of premature mortality in patients with rheumatoid arthritis (RA). It is the result of an accelerated atherosclerotic process. Both RA and atherosclerosis are complex polygenic diseases. Besides traditional CV risk factors and chronic inflammation, a number of studies have confirmed the role of genetic factors in the development of the atherogenesis observed in RA. In this regard, besides a strong association between the *HLA-DRB1*∗*04* shared epitope alleles and both endothelial dysfunction, an early step in the atherosclerotic process, and clinically evident CV disease, other polymorphisms belonging to genes implicated in inflammatory and metabolic pathways, located inside and outside the HLA region, such as the 308 variant (G > A, rs1800629) of the *TNFA* locus, the rs1801131 polymorphism (A > C; position + 1298) of the *MTHFR* locus, or a deletion of 32 base pairs on the *CCR5* gene, seem to be associated with the risk of CV disease in patients with RA. Despite considerable effort to decipher the genetic basis of CV disease in RA, further studies are required to better establish the genetic influence in the increased risk of CV events observed in patients with RA.

## 1. Introduction

Many epidemiological studies have reported in a consistent way an increased risk of nearly all forms of cardiovascular (CV) disease, a higher prevalence of subclinical atherosclerosis, and an increased CV mortality among rheumatoid arthritis (RA) patients [1, 2]. However, traditional CV risk factors, such as hypertension or diabetes mellitus, are not substantially different in RA compared to general population [3, 4]. Therefore, these risk factors may not account for the difference in risk between RA and the general population, and RA-associated factors, such as systemic inflammation, must be the main contributors to the observed gap [5].

Both RA and atherosclerosis are chronic inflammatory diseases [6, 7] that exhibit similar pathophysiological mechanisms [8], and that display a strong genetic component of susceptibility [9–11]. RA has an estimated heritability of up to 60% [12] and CV disease in the general population of up to 30–60% [13]. Besides, a specific genetic background may contribute to the development of both diseases [14].

Subclinical atherosclerosis is present in patients with RA [15, 16]. Noninvasive surrogate markers of atherosclerosis, such as the presence of endothelial dysfunction (established as an impaired flow-mediated endothelium-dependent vasodilatation FMD by brachial ultrasonography) or an abnormally increased carotid intima-media (cIMT) wall thickness and the presence of carotid plaques in the carotid artery (disclosed by carotid ultrasonography) are useful tools to establish a subgroup of RA patients with high risk of CV events [15–18].

Multiple variants in multiple genes have been investigated regarding their association with clinical and subclinical CV disease, traditional CV risk factors, and CV mortality.

## 2. HLA and Related Genes

Alleles from the *HLA-DRB1* gene are not only risk factors for RA but also for CV disease (especially the HLA-DRB1 *0404 allele) [19] (Table 1). Carriers of two copies of the shared epitope (SE) alleles also were associated with about a two-fold increase of mortality from CV disease [19, 20], particularly from ischemic heart disease (IHD) [21]. When specific SE genotypes were analyzed, the HLA-DRB1 *01/*04 combination conferred the highest risk of mortality from CV disease, regardless of the duration of the disease [19–21].

Also, this combination resulted as predictive of mortality due to IHD independently of the inflammatory burden [21]. Furthermore, HLA-DRB1 *04 SE alleles, in particular HLA-DRB1 *0404, were associated with endothelial dysfunction, manifested by an impaired FMD [22, 49] and presence of atherosclerotic plaques [23]. An interaction between alleles of the *HLA-DRB1* gene and other CV risk factors, such as the presence of anti-CCP (which seems to be associated with the presence of preclinical atherosclerosis [50]) has been observed. The combination of carrying two copies of the SE alleles, in the presence of anti-CCP and with a smoking background, appears to confer a very high risk of premature death from CV disease [20].

MHC class II genes expression is regulated by the class II transactivator [51], a protein that acts as a scaffold for the assembly of various transcription factors. This protein is expressed by cells from the atherosclerotic plaque, playing an important role in its development and complication [52]. Two variants of this gene were analyzed by our group, regarding their association with CV disease in two pooled cohorts of Spanish RA patients of Caucasian ancestry: the polymorphism located at position −168 (A > G; rs3087456), previously associated to a lower expression of *MHC2TA* and to a higher risk both of RA and IHD [53], and the variant rs4774, located at position +1632 (G > C; amino acid substitution, glycine to alanine, at codon position 500). Neither of them, either isolated or in combination, showed a significant association with a higher risk of clinical or subclinical CV disease [24].

## 3. TNF Superfamily Genes

TNF-*α* is an important proinflammatory cytokine, with a central role both in RA and atherosclerosis. Two variants in the promoter region of the gene have been analyzed, located at position −308 and −1031.

Regarding the −308 variant (G > A, rs1800629), the minor allele C has been associated with enhanced spontaneous or stimulated TNF-*α* production in both *in vitro* and *in vivo* [54, 55]. Our group observed a significant association of this minor allele with a higher rate of CV events, in two pooled cohorts of RA Spanish patients of Caucasian ancestry [25], only in those subjects carrying at least a copy of the SE, even after adjustment for sociodemographical and traditional CV risk factors. When the influence of this polymorphism in subclinical CV disease was analyzed, although the homozygote for the minor allele showed higher and lower mean values of two surrogate markers of subclinical atherosclerosis, cIMT and FMD, respectively, no statistical significant differences were observed.

The variant located at the −1031 position (T > C, rs1799964) has not shown influence in the transcription activity of the gene [56], neither has been associated to a higher risk of CV disease. However, this polymorphism has been associated with a more proatherogenic lipid profile [57].

Polymorphisms located in the *LTA* gene (that codes for the TNF-*β* or lymphotoxin *α*) have also been studied. This cytokine has been implicated in the early stages of the vascular inflammatory process [58], inducing expression of leukocyte adhesion molecules (LAMs) in endothelial cells. Also, in mice models, its concentration has been correlated with plaque size [59]. The variant located at position +252 (A > G) of the *LTA* gene has been associated with a higher risk of IHD in a cohort of UK Caucasian RA patients [26], with independence of traditional CV risk factors, RA characteristics, and medication.

Galectin-2 is a soluble beta-galactoside binding lectin, that interacts with TNF-*β* and it is required for a correct secretion of the latter into the medium [60]. The polymorphism analyzed regarding its association with CV disease is located in intron 1, at position +3279 (C > T) and has been associated to a decreased transcriptional activity of the *LGALS2 *gene. Although this variant showed association to IHD in general population [60], no association was observed in a cohort of UK Caucasian RA patients [26]. However, the minor allele T showed a significant association, even after being adjusted by confounders, with a lower value of diastolic blood pressure [61].

A polymorphism located in the TNF-*α* receptor II (*TNFRSF1B*) was also studied, located at position +676 (T > G; substitution of methionine for arginine at codon position 196). No association neither with IHD nor with stroke in a cohort of Sweden Caucasian RA patients was observed. However, this variant showed a significant association with a higher risk of hypertension in the same study [27]. 

## 4. Cytokine and Chemokine Genes

IL-6 is a cytokine with pleiotropic and redundant actions. IL-6 is also a major regulator of the synthesis of acute-phase reactants by the liver [62]. This cytokine is produced by a wide variety of cells, and it is involved in both RA and atherosclerosis pathogenesis [63, 64]: circulating IL-6 concentration has been associated with endothelial activation and treatment with traditional disease modifying antirheumatic drugs, resulting in a suppression of IL-6, has been associated to a reduced endothelial activation in patients with RA [65, 66]. Three polymorphisms of this gene were studied regarding their association with CV disease in RA. With respect to the variant located in the 5′ flanking region at position −174 (G > C; rs1800795), previously associated with higher transcriptional activity and increased serum levels of IL-6 [67] in general population, the results have been controversial. A study found a significant association with CV disease [28], adjusting for traditional CV risk factors, but not with a higher risk of hypertension [68], in a relatively small (*n* = 383) cohort of UK Caucasian RA patients [28]. However, in assessing a larger cohort of patients (*n* = 1250), our group did not observe any significant association with clinical CV disease in RA Spanish patients [29], although the minor allele was associated with impairment of FMD in a subgroup of RA patients without clinically evident CV disease [30].

The other two polymorphisms studied (rs2069827 and rs2069840) showed no significant association in the same Spanish Caucasian cohort [29].

Variants located in the two subunits forming the IL-6 receptor were also studied. rs2228145 is located at position +1510 of the *IL6R* gene (A > C; substitution of aspartame for alanine at codon position 358), and its minor allele is associated to higher plasma levels of the soluble form of the receptor [69, 70] and a protective effect regarding CV disease in the general population [71]. However, our group did not observe any statistical association with clinical or subclinical CV disease [31].

In keeping with the above, the polymorphism rs2228044 located at position +755 of the *IL6ST* or *GP130* gene (C > G; substitution of glycine for arginine at codon position 148) was also analyzed in our RA cohort. However, no significant association with clinical or subclinical CV disease was found [31].

The macrophage migration inhibitory factor (MIF) is a cytokine involved in the pathogenesis of RA and atherosclerosis [72]. Two variants of *MIF* were studied regarding their potential association with CV disease: a tetranucleotide repeat element starting at position −794 (CATT_5-8_) and a single-nucleotide polymorphism at position −173 (G > C), both of them were previously associated with higher plasma MIF levels [73]. However, neither of them [32, 33] showed a significant association to CV disease, in two Caucasian RA cohorts from The Netherlands and Spain. The *MIF* −173 variant was not associated to surrogate markers of subclinical CV disease (endothelial dysfunction or abnormally increased cIMT) either [33].

CCR5 is a chemokine receptor that could be potentially involved in the pathogenesis of both RA and atherosclerosis [74], with a proinflammatory effect. The polymorphism analyzed regarding its association with CV disease was a deletion of 32 base pairs (rs333; with starting position at +676) that leads to a truncated nonfunctional receptor [75]. Consequently, in individuals homozygous for the deletion, CCR5 is removed from the cell surface [76], while heterozygous expresses 20% to 30% of the wild-type normal levels [77]. The protective effect of this deletion regarding CV disease in the general population has not always been confirmed [78, 79]. However, in RA population, our group observed a protective effect of the minor allele [34]. Regarding subclinical atherosclerosis, this variant was associated to a higher (normal) value of FMD but no association with cIMT.

The chemokine CCL21 has also been implicated in the pathogenesis of both RA and atherosclerosis [80, 81]. A polymorphism from this gene (rs2812378) was associated to a higher CV and all-causes mortality risk, in a UK chronic arthritis, including RA, cohort [35]. 

CXCL12, also known as stromal cell-derived factor-1, highly expressed in the atherosclerotic plaque [82], has been also studied by our group, owed to the recently association of the rs501120 (T > C) polymorphism with CV disease in Caucasians in a recent genome-wide association study (GWAS) [83]. However, we did not observe any significant association with clinical or subclinical CV disease [36].

## 5. Adipocytokines

Adipose tissue secretes a variety of proteins known as “adipokines” or “adipocytokines” that influence a variety of processes, including metabolism, immunity, and inflammation [84]. Besides, clinical obesity measures, such as body mass index and waist-to-hip ratio, have been recently shown to predict the presence of atherosclerosis in patients with RA from a developed population [85]. 

At present, the most extensively studied adipocytokines are resistin, adiponectin, leptin, and visfatin.

Resistin plays a proinflammatory role [86]. In RA, a strong correlation between resistin serum levels and markers of inflammation, such as C- reactive protein or erythrocyte sedimentation rate, has been observed [87]. The polymorphism rs1862513, located in the promoter region (C > G; position −420), has previously been associated to a higher risk of cerebrovascular disease in type 2 diabetes mellitus patients, both in Asian [88] and Caucasian [89] populations. No association with coronary arterial disease [90], nor with subclinical atherosclerosis was observed [91]. Regarding RA, our group did not observe a significant association with a higher risk of CV disease [37]. Although not included in the published paper, this polymorphism showed no association with the risk of cerebrovascular ischemic events. Regarding subclinical CV disease, the carriers of the minor allele showed a nonsignificant lower FMD and higher cIMT [37]. 

Adiponectin exerts at the vascular level an anti-inflammatory and antiatherogenic role [92], with lower serum levels in subjects with CV disease [93]. However, at the joint level, it exerts proinflammatory effects [94], with a correlation between radiologic joint damage and adiponectin serum levels [95]. We have previously reported that in patients with severe RA undergoing anti-TNF-alpha therapy high-grade inflammation was independently and negatively correlated with circulating adiponectin concentrations whereas low adiponectin levels clustered with metabolic syndrome features that reportedly contribute to atherogenesis in RA [96]. Taking these data into account, two variants of the *ADIPOQ* gene were analyzed by our group regarding their potential association with CV disease. However, none of them was found to be associated with adiponectin plasma levels [97], despite the major genetic regulation in adiponectin synthesis. rs266729, located in 5′UTR (C > G; position −11377), had been associated with a higher risk of type 2 diabetes mellitus in Caucasians [98], and to a higher risk of CV disease, both clinical (in Chinese [99] and in nondiabetic Caucasian subjects [100, 101]) and subclinical (in Caucasian populations) [100, 102]. On the other hand, rs1501299 (G > T; located at position +276 in the second intro of the gene), its minor allele, has been associated to a lower risk of CV disease in Caucasian subjects of south European ancestry [103] and in type 2 diabetes mellitus patients [104], but it showed no association with subclinical CV disease [100]. In our RA cohort, these two variants were not associated with clinical or subclinical atherosclerosis, either when their influence was analyzed isolated or in combination [38]. 

Leptin is a nonglycosylated peptide hormone, encoded by the gene *LEP*, with proinflammatory activities [105], and higher serum levels both in RA and atherosclerosis [106, 107]. In our patients with RA undergoing anti-TNF-alpha therapy, we disclosed a positive correlation between body mass index of RA patients and baseline serum level of leptin [108]. Leptin serum levels were unrelated to disease activity but constituted a manifestation of adiposity in patients with severe RA [108]. Our group analyzed the role of a polymorphism, previously associated with leptin levels [109] and obesity [110], located at position +19 (G > A; rs2167270) in the risk of CV disease [39]. In our study, no significant association was observed with clinical or subclinical CV disease [39].

Visfatin is produced by visceral adipose tissue and immune cells, with proinflammatory and proatherogenic proprieties [111, 112]. The variants rs9770242 (T > G; located at −1001) and rs59744560 (G > T; position −948) were analyzed by our group, owed to their previously reported association with insulin resistance and a proatherogenic lipid profile [113, 114]. However, in our patients with severe RA circulating visfatin levels are unrelated to disease activity, adiposity, or metabolic syndrome [115]. Similarly, in our series of RA patients the visfatin polymorphisms discussed above did not show any significant association, either analyzed isolated or in combination, with clinical or subclinical CV disease [40]. 

## 6. Nitric Oxide Synthase Genes

One of the primary causes of initiation of atherosclerosis is endothelial dysfunction, which leads to altered nitric oxide synthase (NOS) function and NO synthesis [116]. Both the endothelial NOS (eNOS, NOS3) and the inducible isoform of NOS (iNOS, NOS2) have been studied in connection to CV disease. The variant located at exon 7 of the *NOS3* gene (position +5557; G > T), leading to a glutamate to aspartate substitution at codon position 298, was not associated to a higher risk of CV events [41]. The functional effects of this variant in the enzymatic activity are uncertain [117]. On the other hand, two variants of the *NOS2A *gene located in the promoter region were analyzed: the microsatellite CCTTT (with a influence in the gene transcriptional activity [118]) and the single nucleotide polymorphism at position –786 (C > T). Neither variants shows a significant association with the risk of CV disease [41]. However, in carriers of the HLA-DRB1 *0404 allele, these three variants showed association with CV disease [41], suggesting an interaction among different genes.

## 7. Genes Involved in the Haemostatic Process

The plasminogen activator inhibitor type 1 (PAI-1) is the primary physiologic inhibitor of plasminogen activation in blood [119]. PAI-1 overexpression may compromise normal fibrin clearance mechanisms and promote pathological fibrin deposition and thrombotic events. A polymorphism of this gene, located in the promoter region (at starting position –675, 4G/5G), has previously been associated to CV disease and venous thrombotic episodes [120] in the general population. In RA patients, this variant has also been associated with IHD in a cohort of Sweden patients [27]. 

The fibrin stabilizing factor, or Factor XIII, has a crucial role in blood coagulation and fibrinolysis, stabilizing the fibrin clot and making the clot more lysis resistant [121]. A variant located at exon 2 (amino acid change from a valine to leucine at codon position 34), previously associated to a decreased clot formation and lower risk of IHD [122], was analyzed regarding its influence in the risk of CV disease in a cohort of Swedish RA patients. However, no differences were observed [27]. 

Fibrinogen plays a major role in arteriosclerosis and thrombosis [123]: increased plasma levels are an independent risk factor for CV diseases [124]. The polymorphism located at position –455 (G > A) showed no association with a higher risk of CV disease in RA patients [27], despite its association with higher plasma levels [123]. 

## 8. Other Genes

Regarding other variants strongly associated to RA different from those located in the HLA region, such as *PTPN22*, *STAT4* and *TRAF1/C5*, none of them showed a significant association with clinical or subclinical CV disease in a cohort of RA Spanish patients of Caucasian ancestry [42]. In keeping with that, *TRAF1/C5* polymorphism was not associated with a higher incidence of mortality due to CV disease in another two Caucasian cohorts [125].

The 5,10-methylene tetrahydrofolate reductase (MTHFR) is an enzyme that catalyzes the transformation of methionine to cysteine. The lack of this enzyme leads to the accumulation of homocysteine, an independent nontraditional risk factor for CV disease [126]. Two variants associated to a lower enzyme activity and higher homocysteine plasma levels [127, 128] were analyzed by our group. The polymorphism rs1801133 (C > T; position +677; amino acid substitution alanine to valine at codon position 222; exon 4) showed no significant association. Nevertheless, the variant rs1801131 (A > C; position +1298; amino acid change from glutamine to alanine at codon position 429; exon 7) was associated with a higher risk of CV disease and endothelial dysfunction manifested by lower values of FMD [43].

Neovascularization is an important process in the development and stabilization of atherosclerotic plaques [129]. One of the most important pro-angiogenic factors is VEGF. Its expression can be stimulated through both hypoxia [130] and proinflammatory cytokines [131]. Several polymorphisms have been described within the *VEGFA* promoter and 5′UTR regions, which regulate its expression at the posttranscriptional level [132]. We selected two variants with functional relevance: rs1570360 located within the *VEGFA* promoter (G > A; position −1154) and rs2010963 located within 5′UTR (G > C; position −634), both associated to lower *VEGFA* transcription [132]. Also, the latter has been associated to higher serum VEGF levels [133]. The major allele C of the −634 variant has been associated to Behçet disease [134], giant cell arteritis [135] and the presence of severe ischemic complications in the latter [136], and clinical CV disease but only in type 2 diabetes mellitus subjects [133]. However, the −1154 polymorphism has not shown association with CV disease [137]. In our study, we did not observe significant association between any of these two *VEGFA* polymorphisms and clinical or subclinical CV disease in RA patients [44].

*GHSR* codes for the receptor of the growth hormone secretagogue, ghrelin. Besides its metabolic and endocrine effects [138], this receptor also mediates anti-inflammatory [139] and antiatherogenic [140] effects through its expression in immune and vascular cells. 

In RA patients with severe disease undergoing anti-TNF-alpha therapy, we found an increase in ghrelin concentrations upon TNF-*α* blockade that was associated with reductions in P-selectin, a biomarker of endothelial activation that predicts CV event rates [141]. Three polymorphisms were analyzed by our group rs512692, located in the 5′ UTR region, rs509035, located in the intron, and rs2922126, located in the promoter region. Previously, these 3 variants had showed no association with RA [142]. Regarding CV disease, both rs509035 and rs512692 were associated to IHD in the Caucasian general population [143], while rs512692 was not. In our study, we found no association with clinical or subclinical CV disease for any of the variants [45].

Toll-like receptor 4 (TLR4) plays a key role in the activation of innate immune responses and has been implicated in the initiation, progression, and plaque destabilization stages of atherosclerosis [144]. Our group studied the role of the *TLR4* gene polymorphism rs4986790 (position +896; A > G; amino acid substitution from aspartame to glycine at codon position 299), previously associated with an attenuated signaling leading to a dampened response to LPS [145] and with a lower risk of proatherogenic metabolic traits [146]. However, in a German cohort of Caucasian ancestry, this variant did not show association with a lower risk of IHD. In keeping with that, in our population, we did not observe any significant association with a lower risk of clinical nor subclinical CV disease [46].

Low-molecular-weight phosphotyrosine phosphatase (encoded by *ACP1*) plays a key role as regulator of signalling pathways in receptor-stimulated immune cells, in growth factor regulation, in cellular adhesion, and in T-cell development and lymphocyte activation [147]. Three common alleles have been described in Caucasian populations (ACP1*A, ACP1*B, ACP1*C), tagged by two SNPs: rs7576247 (A > G; amino acid substitution from arginine to glutamine at codon position 105; exon 6) and s11553742 (C > T; synonymous polymorphism at codon position 44; exon 3). Another two variants, previously associated to quantitative traits related to type 2 diabetes mellitus [148], were also studied by our group: rs10167992 and rs3828329. We observed that the minor allele of the rs11553742 variant was associated to a significant higher risk of CV disease. Likewise, the classic allele ACP1*C (defined by the presence of the minor allele of the rs11553742 polymorphism and the major allele of the variant rs7576247) also showed a significant association with a higher risk. Both associations remained after adjustment by sociodemography and tradition CV risk factors [47].

Based on the results of previous GWAS [149], a polymorphism located in the region 1p13.3, near the gene *PSRC1* (proline/serine-rich coiled coil protein 1), was analyzed in our group regarding it role in subclinical CV disease. In accordance with those data, we observed a significant association between the minor allele and lower values of FMD [48].

## 9. Conclusions

In the last years, an important advance in the knowledge of the genetic basis of CV disease has taken place, both in general population and in RA patients. However, in aggregate, the discovered variants explain only a small fraction of the heritability of CV disease (estimated to be up to 30–60% in the general population [13]), probably in part due to the limited power of previous studies to discover effects of modest size. Until now, research performed in RA patients was based on hypothesis-driven candidate gene association studies, such as the ones performed in this work. This approach provides a focused view of genomic regions of interest, allowing targeting putative functional variant. Also, this design is especially useful when allele frequencies are low, effect sizes are small, or the study population is limited [150]. However, this kind of approach has its own pitfalls, such as the lack of replication of results, presence of false positive results, and little account for genetic heterogeneity. To overcome these limitations, among other things, it would be necessary to increase the number of individuals, in both cases and controls. To that end, it would be necessary the joint collaboration of different groups to replicate the results in different populations. Also, pooling various cohorts would allow improving the detection of modest genetic effects. 

Taking into account the influence of different genetic markers in the risk of CV disease and CV mortality, we consider that it is maybe the time to start routinely assessing some of these markers in order to identify those RA subjects at very high risk of developing CV disease. for example, we propose to assess the *HLA-DRB1* genotype at the moment of RA diagnosis, due to its repeated association with higher risk of CV mortality, especially in those patients who are anti-CCP-positive and/or current smokers. Besides, these patients at higher risk would probably benefit from a thorough and more intense management of their classic CV risk factors, as, in example, those subjects with a previous CV event. 

A better understanding of the genetic basis of CV disease in RA will grant a better comprehension of the signaling pathways and the knowledge of different molecules implicated in the pathogenesis of this condition. The results derived from these studies will be useful in the discovery of new therapeutic targets and in the development of new drugs that aim to decrease the CV mortality observed in patients with RA.

## Figures and Tables

**Table 1:** Summary of the major genetic studies regarding clinical and subclinical cardiovascular (CV) disease risk and CV mortality in RA patients.

Study	Population	Gene/polymorphism	Controls/cases	Case definition	Results
Gonzalez-Gay et al. [19]	Caucasians from Spain	*HLA DRB1*	143/39	CV mortality, CV disease	*HLA DRB1 * ^ ∗^0404 allele is associated to an increased risk of CV event and CV mortality.
Farragher et al. [20]	Caucasians from the UK	*HLA DRB1*	†	CV mortality	*HLA DRB1 * ^ ∗^01 and 04 are associated to higher CV mortality risk. 2 copies of the SE are associated to a higher CV mortality in RF+ or anti-CCP+ patients. In the latter group, the risk is higher in smokers.
Mattey et al. [21]	Caucasians from the UK	*HLA DRB1*	767/53 CV deaths	IHD mortality	*HLA DRB1 *SE genotypes are associated to higher IHD mortality risk, especially ^∗^0101/^∗^0401 and ^∗^0404/^∗^0404.
Gonzalez-Juanatey et al. [22]	Caucasians from Spain	*HLA DRB1*	55 cases	FMD	*HLA DRB1 * ^ ∗^04 alleles, especially the ^∗^0404/^∗^0404 genotype, are associated to lower FMD.
Rojas-Villarraga et al. [23]	Colombian, ethnicity not clarified in the paper	*HLA DRB1*	140 cases	FMD, cIMT	*HLA DRB1 *SE genotypes are associated to the presence of AP.
Garcia-Bermudez et al. [24]	Caucasians from Spain	*MHC2TA* rs3087456, rs4774	1069/233	CV disease FMD, NTG, and cIMT	No association.
Rodríguez-Rodríguez et al. [25]	Caucasians from Spain	*TNFA* rs1800629	494/93	CV disease FMD, NTG, and cIMT	Minor allele is associated to higher rate CV events, especially in subjects carrying at least a copy of the SE.
Panoulas et al. [26]	Caucasians from the UK (95.9%)	*LTA* + 252 (A > G) *LGALS2* + 3279 (C > T)	301/87	CV disease	*LTA* minor allele is associated to higher risk of MI and HF.
Årlestig et al. [27]	Caucasians from Sweden	*TNFRSF1B* + 676 (T > G)	354/113	IHD, stroke	No association.
Panoulas et al. [28]	Caucasians from the UK	*IL6* rs1800795	295/88	CV disease	Carriers of the minor allele are associated to a higher risk of CV disease.
López-Mejías et al. [29]	Caucasians from Spain	*IL6* rs1800795, 2069827, rs2069840	1030/220	CV disease	No association.
Palomino-Morales et al. [30]	Caucasians from Spain	*IL6* rs1800795	311 cases	FMD	Carriers of the minor allele are associated to lower FMD values.
López-Mejías et al. [31]	Caucasians from Spain	*IL6R* rs2228145, rs2228044	1030/220	CV disease FMD, NTG, and cIMT	No association.
Radstake et al. [32]	Caucasians from The Netherlands	*MIF* −794 (CATT5–8), −173 (G > C)	241/14	CV disease	No association.
Palomino-Morales et al. [33]	Caucasians from Spain	*MIF* −173 (G > C)	249/44	CV disease FMD, NTG, and cIMT	No association.
Rodríguez-Rodríguez et al. [34]	Caucasians from Spain	*CCR5* rs333	558/87	CV disease FMD, NTG, and cIMT	Minor allele is associated to lower risk of CV disease and a normal FMD value.
Farragher et al. [35]	Caucasians from the UK	*CCL21* rs2812378	‡	CV mortality	Minor allele is associated to a higher CV mortality risk.
López-Mejías et al. [36]	Caucasians from Spain	*CXCL12* rs501120	1083/238	CV disease FMD, NTG, and cIMT	No association.
Rodriguez-Rodriguez et al. [37]	Caucasians from Spain	*RETN* rs1862513	553/115	CV disease FMD, NTG, and cIMT	No association.
Rodríguez-Rodríguez et al. [38]	Caucasians from Spain	*ADIPOQ* rs266729, rs1501299	555/119	CV disease FMD, NTG, and cIMT	No association.
García-Bermúdez et al. [39]	Caucasians from Spain	*LEP* rs2167270	655/118	CV disease FMD, NTG, and cIMT	No association.
García-Bermúdez et al. [40]	Caucasians from Spain	*NAMPT* rs9770242, rs59744560	1035/161	CV disease FMD, NTG, and cIMT	No association.
Gonzalez-Gay et al. [41]	Caucasians from Spain	*NOS3* + 5557; G > T *NOS2A *microsatellite CCTTT, –786 (C > T)	143/39	CV disease	No association. Association of minor allele with higher risk of CV disease in carriers of the *HLA-DRB1 * ^∗^0404 allele.
Årlestig et al. [27]	Caucasians from Sweden	*SERPINE1* –675, (4G/5G) *FGB* −455 (G > A) *F13A1* 34 Val/Leu	354/113	IHD, stroke	4G allele was associated to a higher risk of IHD.
Palomino-Morales et al. [42]	Caucasians from Spain	*PTPN22 *rs2476601 *STAT4 *rs7574865 *TRAF1*/*C5 *rs10818488	532/80	CV disease FMD, NTG, and cIMT	No association.
Palomino-Morales et al. [43]	Caucasians from Spain	*MTHFR* rs1801133, rs1801131	532/80	CV disease FMD, NIT, and cIMT	Minor allele of the rs1801131 variant is associated to higher risk CV disease and lower FMD values.
Rodríguez-Rodríguez et al. [44]	Caucasians from Spain	*VEGFA* rs1570360, rs2010963	548/113	CV disease FMD, NTG, and cIMT	No association.
Rodríguez-Rodríguez et al. [45]	Caucasians from Spain	*GHSR* rs512692, rs509035, rs2922126	543/116	CV disease FMD, NTG, and cIMT	No association.
García-Bermúdez et al. [46]	Caucasians from Spain	*TLR4* rs4986790	1220/261	CV disease FMD, NTG, and cIMT	No association.
Teruel et al. [47]	Caucasians from Spain	*ACP1* rs7576247, rs11553742, rs10167992, rs3828329	1374/229	CV disease	Minor allele of the rs11553742 polymorphism and classic *ACP1 * ^∗^C allele are associated to a higher risk CV disease.
López-Mejías et al. [48]	Caucasians from Spain	*PSRC1* rs599839	128 cases	FMD, NTG	Minor allele is associated to lower FMD.

Anti-CCP: presence of anticyclic citrullinated peptides antibodies, AP: atherosclerotic plaque, cIMT: carotid intima-media thickness, CV: cardiovascular, FMD: endothelial-dependent vasodilatation, HF: heart failure, IHD: ischemic heart disease, MI: myocardial infarction, NTG: endothelial-independent vasodilatation, RF: rheumatoid factor, and SE: shared epitope.

†: the cohort consisted in 1022 subjects with inflammatory arthritis, of whom 751 were diagnosed of rheumatoid arthritis (RA). A total of 76 subjects died of CV causes among the whole cohort. The number of death among the RA patients was not clarified in the article.

‡: the cohort consisted in 2324 subjects with inflammatory arthritis, of whom 1027 were diagnosed of RA. A total of 216 subjects died of CV causes among the whole cohort. The number of death among the RA patients was not clarified in the article.
